# PANDEM-Source, a tool to collect or generate surveillance indicators for pandemic management: a use case with COVID-19 data

**DOI:** 10.3389/fpubh.2024.1295117

**Published:** 2024-03-20

**Authors:** Francisco Orchard, Charline Clain, William Madie, Jessica S. Hayes, Máire A. Connolly, Etienne Sevin, Alexis Sentís

**Affiliations:** ^1^Epiconcept, Paris, France; ^2^School of Medicine, University of Galway, Galway, Ireland; ^3^School of Health Sciences, University of Galway, Galway, Ireland

**Keywords:** surveillance, pandemic preparedness, public health, COVID-19, open data, data collection, data generation, pandemic management

## Abstract

**Introduction:**

PANDEM-Source (PS) is a tool to collect and integrate openly available public health-related data from heterogeneous data sources to support the surveillance of infectious diseases for pandemic management. The tool may also be used for pandemic preparedness by generating surveillance data for training purposes. It was developed as part of the EU-funded Horizon 2020 PANDEM-2 project during the COVID-19 pandemic as a result of close collaboration in a consortium of 19 partners, including six European public health agencies, one hospital, and three first responder organizations. This manuscript describes PS's features and design to disseminate its characteristics and capabilities to strengthen pandemic preparedness and response.

**Methods:**

A requirement-gathering process with EU pandemic managers in the consortium was performed to identify and prioritize a list of variables and indicators useful for surveillance and pandemic management. Using the COVID-19 pandemic as a use case, we developed PS with the purpose of feeding all necessary data to be displayed in the PANDEM-2 dashboard.

**Results:**

PS routinely monitors, collects, and standardizes data from open or restricted heterogeneous data sources (users can upload their own data). It supports indicators and health resources related data from traditional data sources reported by national and international agencies, and indicators from non-traditional data sources such as those captured in social and mass media, participatory surveillance, and seroprevalence studies. The tool can also calculate indicators and be used to produce data for training purposes by generating synthetic data from a minimal set of indicators to simulate pandemic scenarios. PS is currently set up for COVID-19 surveillance at the European level but can be adapted to other diseases or threats and regions.

**Conclusion:**

With the lessons learnt during the COVID-19 pandemic, it is important to keep building capacity to monitor potential threats and develop tools that can facilitate training in all the necessary aspects to manage future pandemics. PS is open source and its design provides flexibility to collect heterogeneous data from open data sources or to upload end users's own data and customize surveillance indicators. PS is easily adaptable to future threats or different training scenarios. All these features make PS a unique and valuable tool for pandemic management.

## 1 Introduction

PANDEM-2 is a Horizon 2020 EU-funded project aiming to develop and demonstrate innovative solutions to strengthen pandemic preparedness and response at the EU level for public health emergencies at subnational, national, EU, and global levels. The IT tools are accessible through an interactive decision support dashboard that encompasses data for disease surveillance from a variety of domains including data from traditional epidemiological surveillance data sources, non-pharmaceutical interventions, contact tracing, and hospital resources, but also data from non-traditional surveillance data sources such as data from social or mass media analysis, participatory surveillance and flights. An integrated epidemiological and hospital resource capacity modeling is also available to support planning and what-if scenarios.

The PANDEM-2 consortium includes partners that cover key aspects of pandemic preparedness and response including six National Public Health agencies in the EU (RKI in Germany, FOHM in Sweden, THL in Finland, INSA in Portugal, NIPH in Romania, and RIVM in the Netherlands) and three first responders organizations (Austrian Red Cross, Italian Red Cross, INEM Portugal), and the Radboud Medical Center in the Netherlands. All the tools developed were designed and validated in close collaboration with the consortium partners and are distributed using open-source licenses.

PANDEM-Source (PS) is an IT surveillance tool to collect and integrate openly available public health-related data from heterogeneous data sources to better support communicable disease surveillance for pandemic preparedness and response. PS's main objective is to identify, map, and integrate pandemic-related data from multiple sources into a coherent pandemic-management database so it can provide all the necessary data to feed the PANDEM-2 dashboard with, when available, near real-time data. Its data model was developed in close coordination with the consortium partners aiming to address the challenge of monitoring the COVID-19 pandemic response, but it is flexible and can be adapted to other diseases or new threats, variables, and indicators by changing source description files without changes in code.

Effective training of public health professionals is an essential element to strengthen pandemic management (1), which is targeted by the PANDEM-2 project by developing training scenarios and simulation exercises (2). During the simulation exercises, the PANDEM-2 dashboard displays realistic information matching a specific designed scenario for training.

Collecting and producing the required data can be a challenge due to the broad scope of information displayed on the dashboard for pandemic management and training. To ensure quality and facilitate processes, PS includes monitoring systems/visualization of data collection by source and features to detect missing data on an initial set of indicators. The data generation process is based on these “initial” indicators to create a realistic full synthetic dataset covering all necessary variables to perform the simulation exercises. These features were used during the PANDEM-2 simulation exercise, which focused on assessing PANDEM-2 tools applied in Public Health Emergency Operation Centers in Germany and the Netherlands during an influenza pandemic scenario. For this scenario the initial set of indicators was produced using the PANDEM-2 modeling tools and included confirmed cases, deaths, and vaccination status by age group for Germany and the Netherlands. Subsequently, PS generated data for the remaining variables in the dashboard for all EU/EEA countries, including subnational level and distribution by age group, sex, and presence/absence of comorbidities. These generated variables encompassed data on contact tracing, hospital resources, and social media analysis trends (sentiment, emotion, and suggestion). After data generation, PS performed the calculations for those indicators that needed to be computed such as bed occupancy, incidence rates, mortality rates, vaccination rates, etc.

In this paper, we will describe PS's features and design with the aim of disseminating its characteristics and capabilities to strengthen pandemic preparedness and response.

## 2 Methods

### 2.1 Identification of relevant variables and indicators for pandemic management

**Figure 1:**
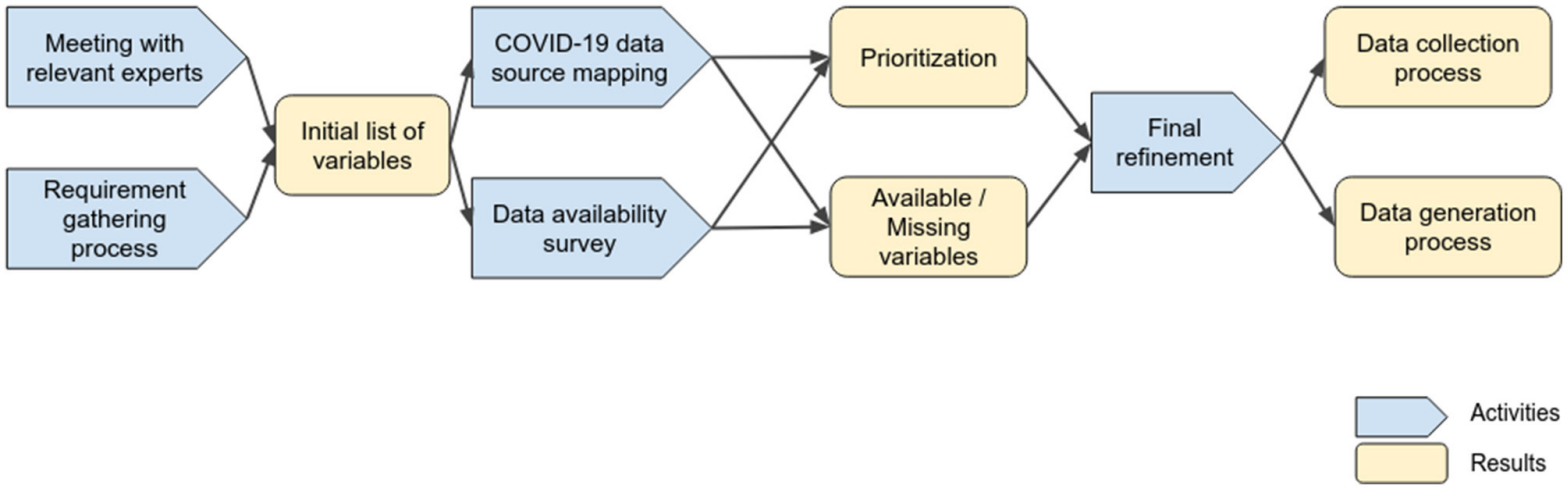
Process followed for identifying pandemic management variables and indicators to be collected or generated.

The PANDEM-2 project commenced 12 months after the onset of the COVID-19 pandemic in the EU. The initial work involved a requirement-gathering process with the European public health agencies and first responders on the PANDEM-2 consortium. We conducted a structured process to identify the most important variables and indicators to be included in a dashboard to address current and future needs for pandemic management. This process is described and discussed in (2) and included the following steps: A web and literature search and meetings with experts, which allowed us to identify relevant data sources for the project as well as which ones were openly available. In parallel, all consortium participants were invited to provide a list of data requirements, variables and indicators according to their ideal dashboard to be used for pandemic management. The list of requirements and meeting outputs were analyzed to produce an initial list of variables that were grouped in different data families. A data survey was distributed to end users to score the relevance for pandemic management and to provide details on their data priorities and availability for all the identified variables focusing on the COVID-19 use case. These outputs were used to accomplish a final refined list of variables taking into consideration PANDEM-2 partners's assessment of importance, priorities, and data availability. We defined an automatic data collection process for variables available in open data sources and data generation for non-available variables. We generated synthetic data for missing relevant variables to showcase the full potential of the PANDEM-2 dashboard and to introduce it as a training resource for pandemic management. Figure 1 schematises this approach by stating activities, results, and dependencies.

### 2.2 Design goals

PS was designed to achieve the following design goals:

- Adding new sources and variables should be possible without changes in code.
- Keep track of the reporting institution and methodology for collecting and computing data.
- Capability of integrating data from a wide variety of sources and formats.
- Automatic data standardization based on the source description having capacity from transcoding from multiple coding schemas, e.g., the region name in local languages to the region code.
- Integration, type, and standardization errors are informed to the data manager.
- The data integration process should be fault-tolerant. Errors during data importing process should not lead to data loss.
- Generating synthetic data with the purpose of using the PANDEM-2 dashboard as a training resource for pandemic management.

### 2.3 Assumptions

In order to generate a generic approach for data integration we defined the following assumptions:

- A variable is a label indicating a general concept. Some variables are computed indicators that can be evaluated using data collected directly from data sources, e.g., incidence rate. Names are previously defined and are associated with a particular definition, the type (numeric or text), coding schema, and/or calculation method.
- Limited variable types: integer, numeric, date, datetime, and string.
- Variables can be grouped into observations and attributes. Observations contain mainly epidemiological information such as “number of cases” or “incidence rate.” Attributes provide extra information or characteristic details associated with a particular observation, e.g., the age group of the observed cases.
- Variables can be tied together as tuples containing a unique measure, a date, a source, and several attributes:

° Confirmed cases:13
° Pathogen: dengue
° Age group: 10–18
° Date: 2021-12-13
° Geo: Brussels
° Source: ECDC

- Time series are built using observation values in time for tuples with the same attributes, e.g., the evolution of confirmed cases for a given age group and city. Indicators can be calculated using functions at the time series level, e.g., the time series for the effective reproduction number (Rt) is obtained based on the time series of the number of confirmed cases over time for a disease and the given population in that geographical location.
- For a given set of attributes and an observation, there can be only a single unique value.
- Data sources provide stable identifiers that can be used for unequivocally retrieving the associated data.
- Data source resources can be read in a tabular format.

### 2.4 Data pipeline design

To tackle the issue of data collection and generation we developed the data labeling schema (DLS), a declarative methodology based on text files, for documenting, standardizing, and integrating surveillance data sources and producing homogeneous and comparable time series. This methodology provides a common approach to address the heterogeneity of formats, sources, and types of data found during the COVID-19 pandemic.

The DLS integration pipeline uses a set of source description files providing information including ownership, how to detect changes to trigger an import, the file format, how to read the files in a tabular format, how to map columns to PS variables, and the applicability of data generation. The list of PS variables contains functions for calculated indicators such as incidence or mortality rate so they can be automatically calculated when the required parameters for its computation are present. Such functions are defined in R language, a widely used language in the domain of epidemiology. Advanced calculations can be performed by integrating third-party algorithms. For instance, social media analysis data are obtained thanks to natural language processing algorithms developed by the University of Galway. A final aggregation step is performed up to country level if not previously provided by the source.

Providing data for training purposes can be a difficult task since not all expected data are available or because the training scenario is completely fictitious. To address this issue, PS includes data generation formulas. When a data source for a training exercise is loaded, PS will automatically detect which variables are missing and will evaluate the synthetic formulas to generate the missing time series. When partitioned data is missing, e.g., deaths by comorbidity, a weighted sample function is applied using probabilities between groups matching the specific scenario. When data is missing for a training exercise, hypothetical estimations are created based on present data. For example, to estimate the number of people traced for contact tracing we used the number of public health workers available and a given capacity to daily contact new confirmed cases, allowing us to simulate system overloading. It should be noted, that here our primary intention was to use these formulas to align with the training scenario previously mentioned, and not to represent accurate epidemiological data estimations. To avoid confusion with real data, time series generated in such a way are tagged in the resulting dataset as “synthetic.”

**Figure 2:**
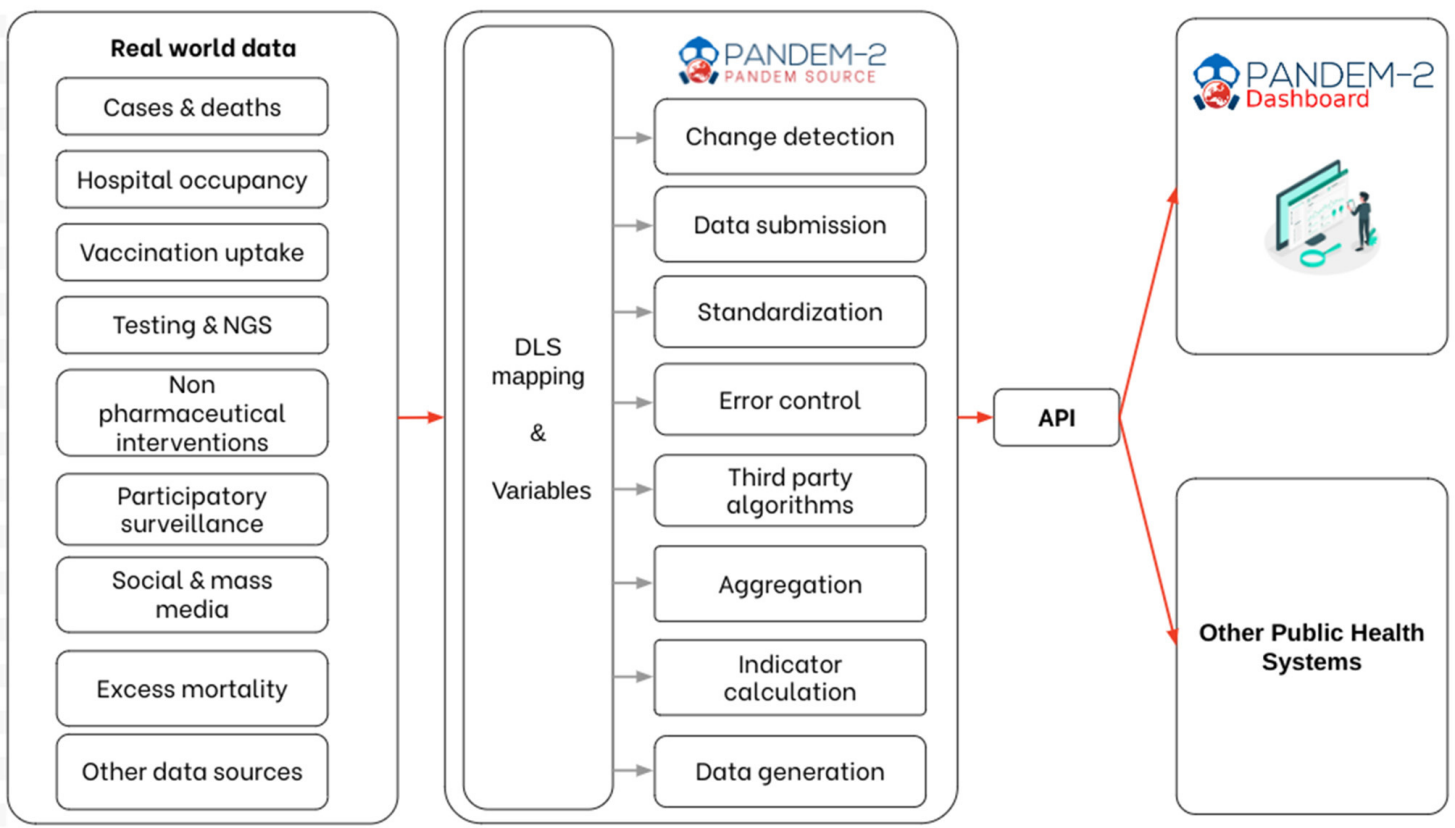
PANDEM-Source (PS) integration pipeline.

The resulting data are stored as JSON files and available using a REST API (3) allowing to query the integration process and obtain the results. Figure 2 shows the entire PS integration pipeline.

## 3 Results

### 3.1 The list of variables

The process of meeting data requirements and defining pandemic management variables produced a list of 29 main variables (Table 1). The complete list of variables is described in (4). Variables are grouped into data families associated with different aspects of pandemic management. Rates and stratification by different attributes are computed for most of the main variables.

**Table 1:** List of PANDEM-Source (PS) variables.

**Data family**	**Main variable**	**Stratification and rates**
Cases	Confirmed cases	By genetic variant, by sex, by presence/absence of comorbidities (presence or absence of any comorbidity), rate in the population (incidence rate)
	Number of notifications	Rate in the population
	Active cases	Rate in the population
	Recovered cases	Rate in the population
	Rt number	
Deaths	Deaths	By bed type (ICU, ward), mortality rate in the population, mortality rate within hospitalized patients
	Excess mortality	
Hospital capacity	Hospitalizations	By bed type, by presence/absence of comorbidities, by presence/absence of comorbidities and bed type, bed occupancy rate, hospitalization rate in the population, hospitalization rate in patitents with/without comorbidities
	Average length of stay	By bed type
	New Hospitalizations (admissions)	By bed type, by presence/absence of comorbidities, new hospitalizations bed occupancy rate, new hospitalizations rate in the population
	Available staff	By staff type (medical doctors, nurses, etc.), rates in the population
	Bed capacity (available beds)	By bed type, bed capacity rate in the population
Syndromic surveillance	Primary care cases	By ILI/ARI and SARI
	Primary care positivity rate	By ILI/ARI and SARI
Testing and lab	Performed tests	By genetic variant, by test type (PCR, antigen test, etc.), positivity rate, testing rate
	Sequenced samples	By mutation, by genetic variant, sequenced rate among tested
Vaccination	Doses injected	By dose number, vaccination rate by dose
	People fully vaccinated	Vaccination rate
Contact tracing	Contact tracing cases	By being already a contact (previously identified as a contact of a case), by reached status (if this person have been contacted), by reached in a day (reached within 24 hours after their test result)
	Contact tracing contacts	By reached status, by reached in a day (reached within 24 hours after contact identification, i.e., same day or day after)
Participatory surveillance	Participants declaring symptoms	
	Number of participants	
	Estimated incidence	
	People seeking health care	By visit type (primary care, emergency, hospital)
Non-pharmaceutical interventions	Implemented_measure	By specific measure (e.g., Contact tracing)
Population studies	Seroprevalence	By study name
	Studied population	By study name
Transport	Number of incoming flights	By country of origin
Social and mass media	Article count	By topic, sentiment, emotion and suggestion
Referential	Population	By subpopulation (age group, sex, etc.)

The complete list of variables is described in (4).

### 3.2 Integrated sources

PS uses a set of source description JSON files containing the necessary information for integrating data and calculating time series. During the PANDEM-2 project, we developed source description files for European Union countries using data from the COVID-19 pandemic. Collected data was limited to the publicly available sources listed here below and excluded any individual case data. We used the data available at the most fine-grained geographical level.

It is important to highlight that PS supports an all-hazard approach (5). Given the emergence of COVID-19 and due to the extensive heterogeneous data required for pandemic management, this proved to be a valuable use-case. However, PS can be easily extended to support other diseases or threats, with their variables and indicators, as well as other geographical scopes.

Sources used to build the PANDEM-2 COVID-19 dataset (4) can be grouped into those used as indicators (or to compute indicators) and those used for standardization. The list of sources implemented are the following:

- Sources used for indicators:

° ECDC COVID-19 datasets (6): Selection of datasets describing the EU members's surveillance and response to the pandemic published by the European Center for Disease Prevention and Control (ECDC).
° COVID-19-Datahub (7): A unified dataset collecting global fine-grained case data on the pandemic surveillance and response. This dataset is used for indicators that are not available at a subnational level on ECDC datasets.
° Our World in Data (8): Our World in Data is a scientific online publication that focuses on global problems. It provides several COVID-19-related datasets. This source was used to obtain excess mortality data since this indicator was missing from previous sources.
° Influenzanet (9): A Europe-wide network to monitor the activity of influenza-like illness (ILI) with the aid of volunteers via the Internet. It is currently operational in 12 countries. Influenza.net also publishes datasets on participants declaring COVID-19 symptoms and healthcare-seeking behavior as well as provides estimated incidence for the participating countries.
° Twitter, via the Panacea Lab COVID-19 tweet collection (10): A large-scale COVID-19 Twitter dataset for open science starting in March 2020. This dataset contains COVID-19-related tweet IDs. We obtained the list of IDs from the data-sharing platform Zenodo.org and downloaded the tweet texts using the Twitter API. The tweets were annotated using Natural Language Processing models to produce dedicated time series by country.
° MediSys (11): The Medical Information System MedISys is an internet monitoring and analysis system developed at the European Commissions Joint Research Center in collaboration with ECs Directorate General for Health and Consumer Protection (DG SANCO) to rapidly identify potential threats to public health using information from the internet. MedISys continuously monitors approximately 900 specialist medical sites plus all the generic EMM news, i.e., over 20,000 RSS feeds and HTML page sites from 7,000 generic news portals and 20 commercial newswires in altogether 70 languages. We generated a connector for Medisys capable of extracting the last 30 days of articles for predefined topics but due to the lack of historic data access, this source could not be included in the COVID-19 dataset.
° OpenSky Network (12): OpenSky Network is a non-profit association that provides open access to flight tracking control data. It was set up as a research project by several universities and government entities with the goal of improving the security, reliability, and efficiency of the airspace. The used dataset is a derived version from the full dataset published on Zenodo covering the period from 2019 to 2022.
° Eurostat (13): Eurostat is the statistical office of the European Union and publishes a wide range of datasets and statistics concerning European countries. We obtained information about available beds and hospital staff resources.
° OECD (14): The OECD (Organization for Economic Co-operation and Development) regularly publishes comparable statistics on numerous subjects. In the case of COVID-19, it was the selected source for obtaining an estimation of the evolution of the number of Intensive Care Unit (ICU) operational beds by country.
° SeroTracker (15): SeroTracker is a dashboard and data platform for SARS-CoV-2 serosurveys. They conduct systematic reviews to track serosurveys (antibodies testing-based surveillance) around the world. Seroprevalence studies results were integrated.

- Sources used for standardization:

° Eurostat (16): The NUTS classification (Nomenclature of territorial units for statistics) is a hierarchical system for dividing up the economic territory of the EU and the UK. Standard region codes and names were obtained.
° Geonames.org (17): GeoNames is a geographical database distributed under the Creative Commons attribution license. It contains over 27 million geographical names. We used this source to extract ISO2 and ISO3 code equivalences and to obtain multilingual aliases for countries in the world and regions in Europe.
° ICD-10-CM (18): The ICD-10 Clinical Modification (ICD-10-CM) is a modification of the ICD-10 (International Classification of Diseases) used as a source for diagnosis codes in the United States of America. This source was used to obtain the list of pathogens.
° ISCO-08 (19): ISCO-08 ISCO-08 is a four-level hierarchically structured classification that allows all jobs in the world to be classified into 436 unit groups. We used this codification to store information about staff resources types in hospitals such as doctors or nurses.
° OurAirports (20): OurAirports is a free site where visitors can explore the world's airports, read other people's comments, and leave their own. This site provides freely available files with the list of world airports.

Since PS's sources are currently in the public domain, there are no particular risks in widely sharing the tool or the produced outputs.

### 3.3 PANDEM-Source pipeline implementation

#### 3.3.1 Source description

The Data Labeling Schema (DLS) is a declarative approach that requires a detailed description of the sources using JSON files in complement with a list of variables, mappings, and indicator formulas. Based on the source description, PS takes care of all transformations to perform the data acquisition, validation, standardization, and, if necessary, the calculation of indicators or data generation. Each data unit goes through the same standardized integration pipeline (Figure 2). This reduces the risk of errors and ensures updated metadata are kept in the final database. Each variable is linked to a unit, a source, and a date of integration, and mapped to a target “pre-defined” PS variable providing a description, a referential (if transcoded was necessary), and associated formulas. If new variables need to be added, the list of variables can be directly modified to include new concepts without changes in code. A CSV file can be easily modified by a data manager allowing complete autonomy on variable definitions, which is a key feature to allow adaptation to unknown or novel threats. The file is publicly available on GitHub.^1^

#### 3.3.2 Data acquisition

The source descriptor file of each source must also define all necessary information to monitor each source, trigger automatic updates when changes are performed on the source, and how to interpret file format to acquire data. Multiple acquisition channels were implemented including git repositories, URLs, and predefined Application Programming Interfaces (APIs). If a new channel is required, the user can provide custom R or Python scripts to perform the data acquisition. PS checks for updates on a regular predefined basis using when-available versioning methods to avoid downloading data to detect changes.

The source descriptor files also define the format of target files, including Excel, CSV, JSON, and XML. Each format has its own formatting properties to interpret the provided data. If the source files are too complex or need to be cleaned before integration, PS supports the usage of dedicated Python scripts to pre-process datasets.

#### 3.3.3 Standardization

A number of well-defined standards (21, 22) are included in PS and the tool automatically monitors and updates these references from public data sources to compute specific indicators, from NUTS, ISO country codes, ICD-10 diseases, or geonames. When input data do not match the expected format or referential data, PS provides a list of integration issues allowing the data manager to visualize and fix them. For instance, if a source provides the number of confirmed cases for an unknown country, the data are ignored and details of integration issues are reported. The user can define mappings using JSON files to support transcoding between different codification systems such as ISO3 country codes or region names to NUTS.

#### 3.3.4 Calculated metrics and indicators

Calculated indicators such as incidence rates and effective reproduction number (Rt) are produced by R scripts included in PS, which can be also modified by any user with basic knowledge of R language. PS proceeds automatically to indicator calculation whenever all necessary parameters are provided by a source. The indicator can also be collected if already computed on the source. Computing indicators is preferred to directly collecting them from data sources to ensure the same methodology is used, thus supporting comparability. Aggregation from the subnational to the national level is also performed automatically.

#### 3.3.5 Data generation

In the list of variable definitions, PS also includes formulas for data generation allowing creating the entire PS datasets from a minimal set of input variables. This data generation feature was designed to generate training datasets that can be used during simulation exercises. This feature was used in the PANDEM-2 Functional Exercise which simulated pandemic Influenza and included two National Public Health agencies (Germany, the Netherlands) supported by all other end user organizations within the PANDEM-2 consortium. The PANDEM-2 modeling tools (23) were used to produce the time series for the number of cases, deaths, hospitalisations, and vaccinations, and PS used its data generation functions for generating plausible data about social media posts, participatory surveillance, contact tracing, public health staff variations, syndromic surveillance, and stratifications by comorbidities and age groups. This feature reduced the amount of effort needed to prepare the data for the simulation exercise.

### 3.4 PANDEM-Source architecture

PS is a Python package that implements the DLS with out-of-the-box definitions for integrating a wide range of indicators from heterogeneous surveillance data sources. It has been published on Pypi^2^, its code is open under the EUPL^3^ license and it is available on GitHub^4^.

**Figure 3:**
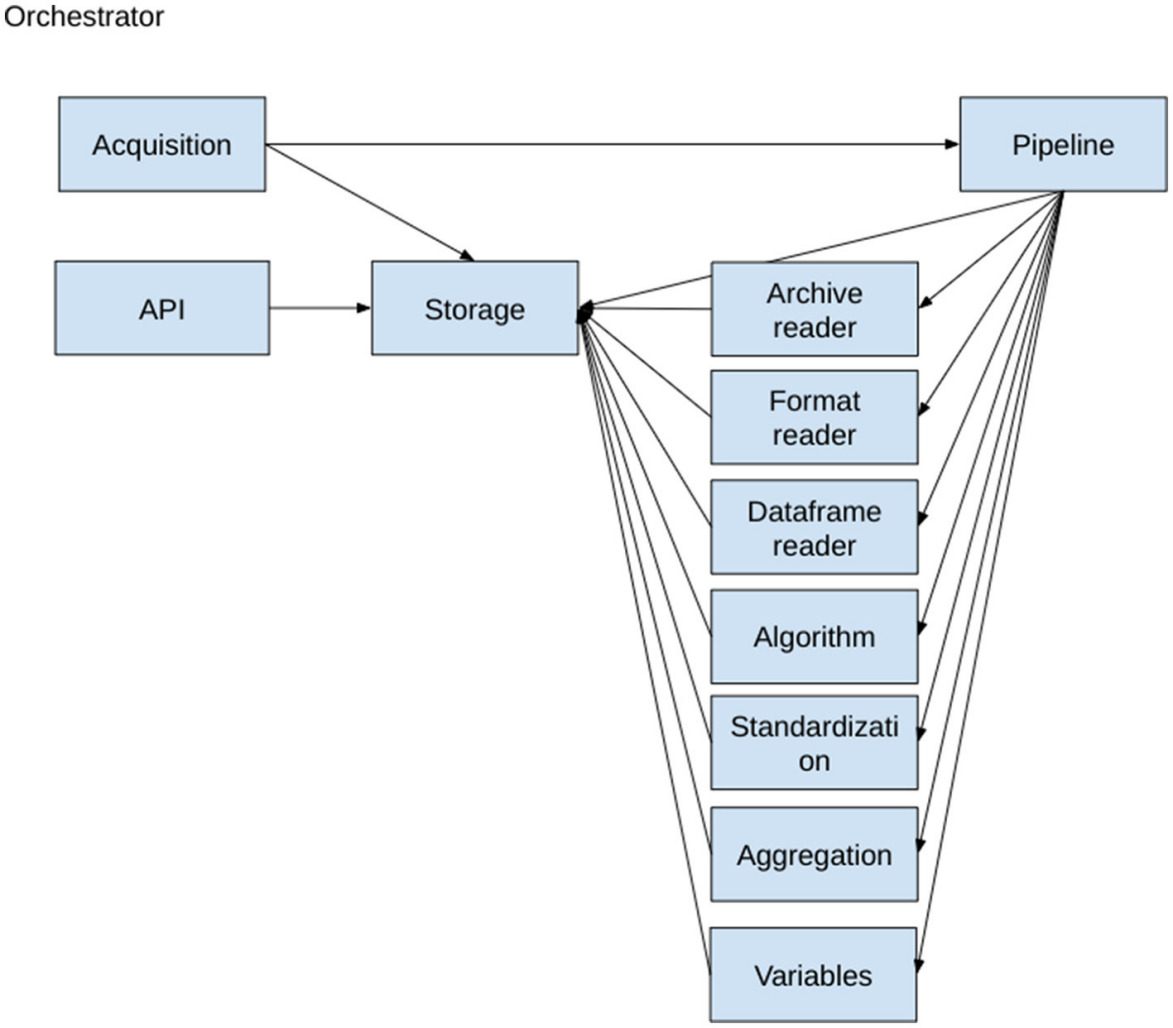
PANDEM-Source (PS) actor dependencies.

PS has been implemented following a microservices architecture using the Actor Model and using the package with pykka.^5^ The actor architecture diagram is shown on Figure 3. Each actor receives messages, processes them one by one, and can send messages to other actors. Messages can be any Python object. This programming pattern allows to achieve a good level of parallelization of tasks while keeping a simple programming model and file access. For external algorithm integration, we have used docker containers and REST APIs. The following classes of actors have been defined:

- **Orchestrator:** Launch actors, manage docker encapsulation, and close actors.
- **Storage**: Keep persistent information of the integration process and process all data storage operations.
- **Acquisition**: Triggers data integration of known data source files.
- **Data pipeline**: Ensures that integration is performed and ensures that the process will run until an end (error, warning, or success).
- **Algorithms:** Execute a particular algorithm during the pipeline.
- **Format readers:** Transform input files into data frames.
- **Dataframe reader:** Transform data frames into a list of non-standard tuples.
- **Standardization:** Transform a list of non-standard tuples into a list of standard tuples.
- **API:** Provides a REST API for accessing public endpoints.
- **Variables:** Reads and writes standardized variable values. Provides necessary mapping information to standardization actor in order to standardize variable values e.g., Country Name = > ISO code.

### 3.5 Integrating social media analysis components

The integration of social media components necessary to classify tweets required the execution of Social Media Analysis (SMA) components developed as part of PANDEM-2. These components were packaged and exposed as an API and utilized a TensorFlow Serving Docker to facilitate its integration. PS includes all necessary parameters to automatically launch the right docker tensor flow server locally if not already running on the configured URL; the only information that is required to run the algorithms is the folder where the models are saved. The models are launched using configuration files, so adding new models does not require changes to its code.

Once the models are running, PS will evaluate any source including the variable *article text*, and add the resulting model outputs as new attributes of the related tuple.

After annotation, the article text is removed for data protection reasons, and PS will calculate the aggregations for each used algorithm and produce the related time series.

### 3.6 Integrating the next generation sequencing simulator

Another external algorithm utilized is the Multiparametric Next Generation Sequencing (NGS) simulator (24). This tool is used to generate realistic time series by variant and mutation not being publicly available. The simulator combines real data from different sources to produce non-available time series such as cases by variants or the number of hospitalizations by age group and vaccination status. The resulting datasets are built using machine learning approaches to find a realistic combination of these variables. This simulator is written in R.

PS uses the simulator, which needs to be installed as an R package. It uses git to locally check if there have been changes on the input files before launching the simulator.

### 3.7 PANDEM-Source python package

PS includes a Shiny^6^ app for validating the integration and visualizing the integrated time series. The ‘data integration dashboard' is structured as follows:

#### 3.7.1 Data integration page

**Figure 4:**
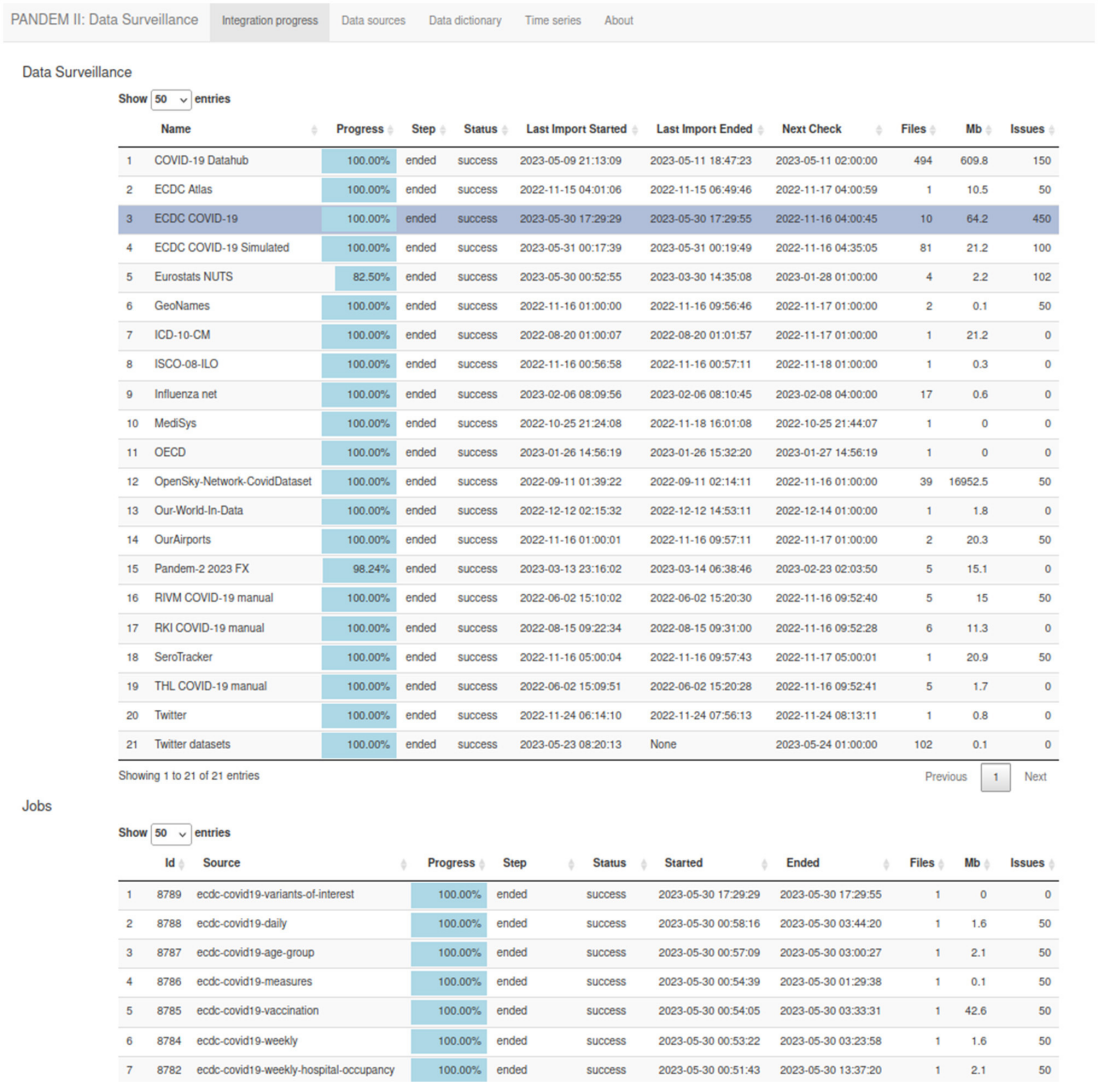
The PANDEM-Source (PS) data integration page.

List of integrated sources showing current integration status, next expected check for changes, the history of data sets collection executed, and issues found (see Figure 4). This page refreshes automatically.

#### 3.7.2 Data sources page

**Figure 5:**
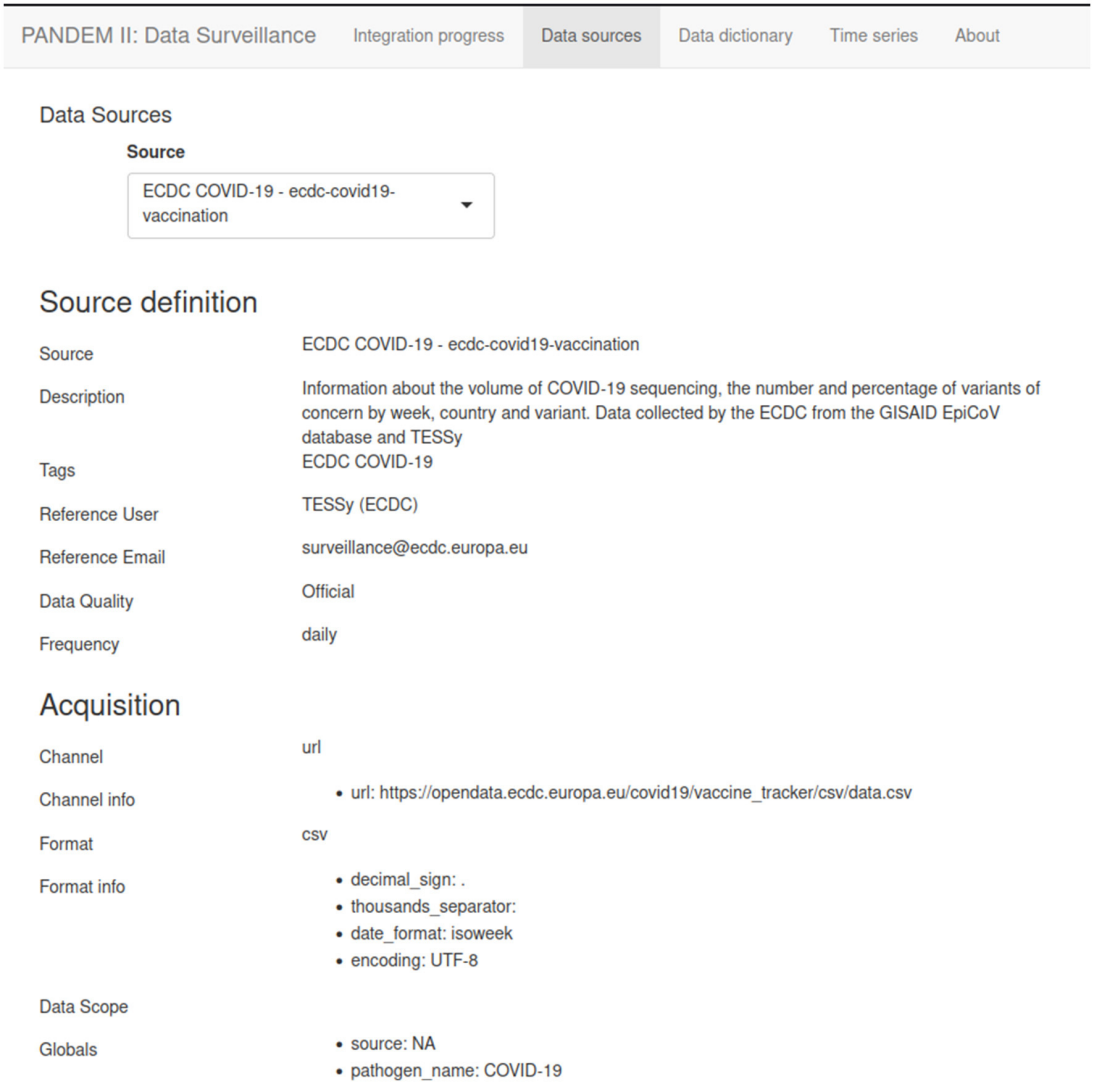
The PANDEM-Source (PS) data source page.

List of defined data sources including information about the source descriptor files such as acquisition channel and variable mappings (see Figure 5).

#### 3.7.3 Data dictionary page

**Figure 6:**
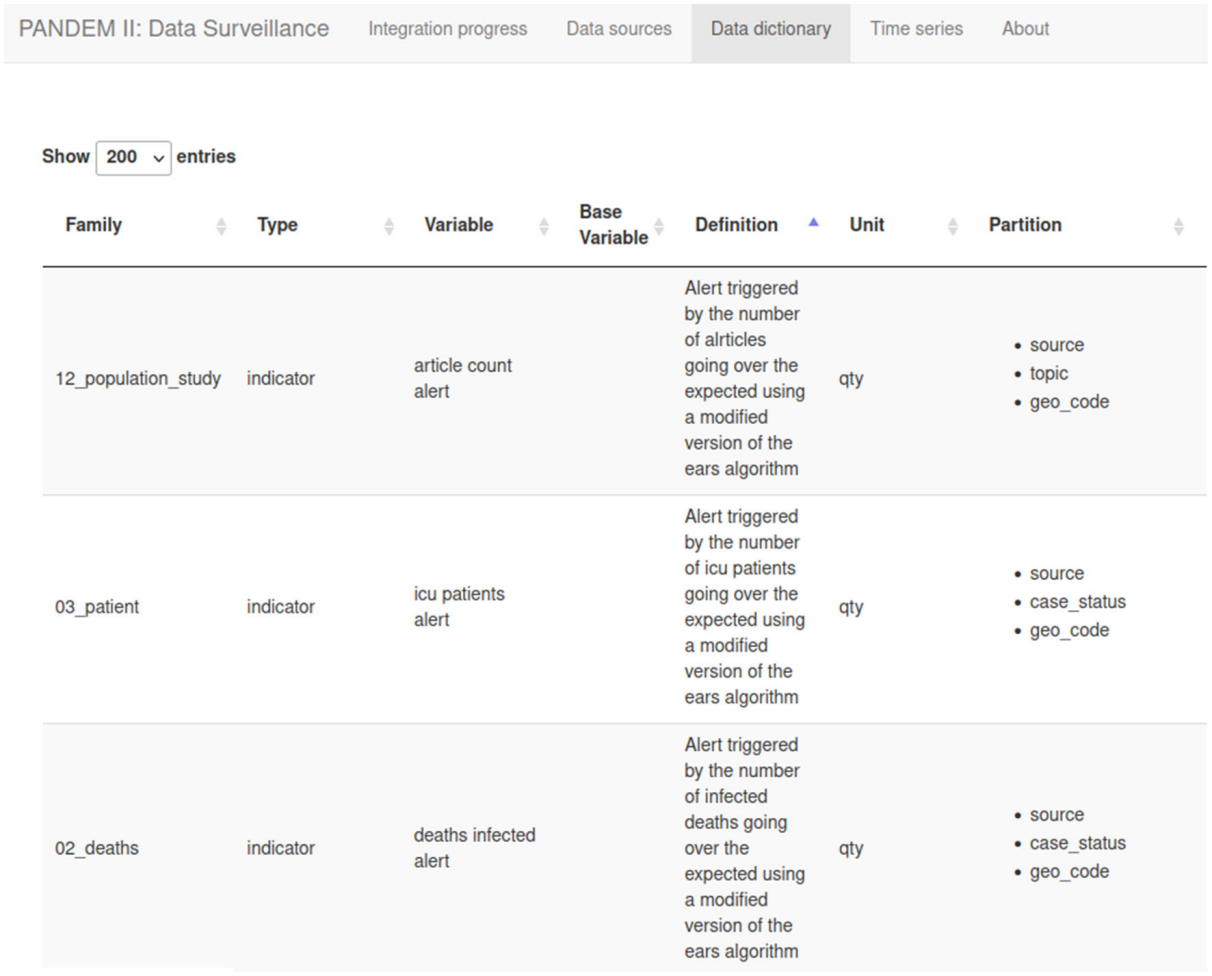
The PANDEM-Source (PS) data diccionary.

The entire list of variables defined on PS including all metadata and formula definitions (see Figure 6).

#### 3.7.4 Time series page

**Figure 7:**
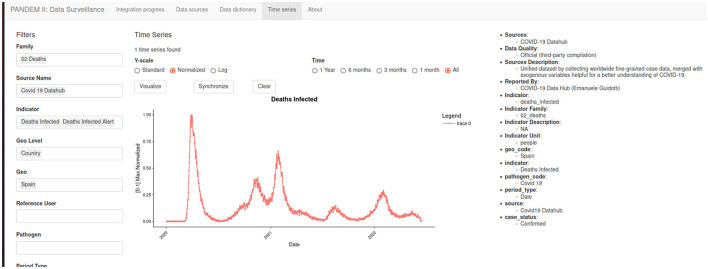
The PANDEM-Source (PS) time series page.

Displays all the integrated time series. A dynamic filter system and the count of matching time series help the user explore and understand the underlying data (see Figure 7). Time series from different sources can be easily compared.

#### 3.7.5 Exporting data

Data processed by PS is available via a REST API. Which can be regularly called to get the most current data.

**Figure 8:**
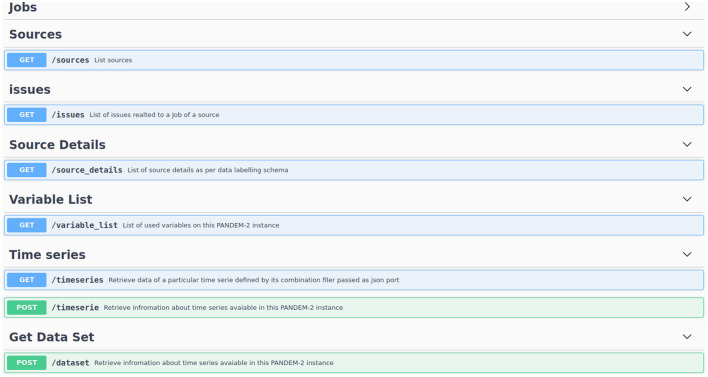
Implemented endpoints of the PANDEM-Source (PS) REST API.

The REST API is also the way of acquiring data for the Shiny^6^ ‘data integration dashboard' so any data on the integration dashboard can be replicated on the PANDEM-2 database. Figure 8 displays the implemented endpoints of the REST API.

## 4 Conclusions

In the context of the COVID-19 pandemic and within the umbrella of the PANDEM-2 project, PS has been developed in collaboration with large variety of health and public health experts to include within the PANDEM-2 dashboard relevant indicators for infectious disease surveillance and to monitor the health response. During the PANDEM-2 simulation exercises, PS and the PANDEM-2 dashboard have proven useful to facilitate training for pandemic management. The design provides flexibility to collect data from open data sources or to upload end users's own data and customize indicators. PS can support public health agencies and first responders in developing their own data collection tools according to their specific training and response needs and reduce the development effort of building them from scratch. The flexibility and easy-to-customize are the main features of PS which are supplemented by the capacity to generate realistic epidemiological synthetic datasets that can be used for training purposes. All these characteristics make PS a unique and valuable tool for pandemic management. To the best of our knowledge, although some data collection tools have been developed during the COVID-19 pandemic (7, 25, 26), PS is unique in terms of flexibility and customization to be adapted to different data sources or diseases or to generate data for training. By its broad approach, it also differs from other initiatives by the heterogeneity of the data sources and collected data. Furthermore, its flexibility allows to quickly adapt to emerging threats which impose new data needs. We believe these characteristics make the tool useful not only for training purposes but also, with further development and adaptation according to the context, to be deployed to support monitoring and managing an emergent epidemic or pandemic.

At the end of the data integration process, PS also allows users to visualize the uploaded data as time series by geographical level or other shared attributes, such as variant or age group, which may facilitate monitoring and validate the surveillance results before being captured in the dashboard (or just to visualize some results from some variables not incorporated in the dashboard). This approach leverages visualization and comparison of any available indicator. For instance, it allows the visualization of social media emotion trends together with other variables such as the evolution of the seroprevalence in a specific population or the evolution of people's opinions to specific public health-related topics or measures which is also based on social media analysis.

PS is a ready-to-use open-source tool allowing pandemic managers to collect and harmonize multiple surveillance data on specific pathogens from traditional and non-traditional publicly available or restricted data sources. It currently collects data for COVID-19 surveillance and response from different domains (cases, deaths, ICU beds occupancy, social media analysis, Lab and NGS data, and non-pharmaceutical interventions) and different data sources such as ECDC or other public health agencies' websites—via the COVID-19 Datahub scripts, Influenzanet, Twitter, MediSYS, etc.

In summary, PS contributes to the pandemic management community through:

• Out-of-the-box data collection for pandemic preparedness and response:

° Flexibility and customization of data collection.
° Data can be visualized in the tool.
° Exploited directly through the PS API.
° Visualized through the PANDEM-2 dashboard.

- Simplifying cross-domain data collection for epidemiological surveillance.
- Allowing foundation for a multi-source multi-threat early warning system.
- Proposing a standard methodology for collecting surveillance data and computing indicators that could support standardization and data sharing among countries.
- Generating data to be used for training purposes.

It is relevant to highlight some potential limitations when using the tool. Firstly, data availability may be a limiting factor. It is possible, as may happen at the beginning of an epidemic or pandemic, that data are scarce. Secondly, we found that, although some data may be potentially available, there are data sharing limitations in different scenarios, such as sharing sensitive data with the general public or with other countries. There may also be restrictions to share personal data for public health surveillance purposes without patient consent. Related to these previous points it is worth mentioning that often the most useful data to respond at the local level is not available or, if available, the data are sensitive due to data protection issues (possibility to identify individuals). Thirdly, the list of data sources required (open or restricted) may substantially change over time which can be explained, among other reasons, because of changes in data providers, data sharing procedures or permissions but also due to shifts of threat or pathogen. Finally, some users may need some training to install and use the tool (it is not always possible to have qualified assistance). Despite these limitations, we consider that PS is a useful tool for pandemic preparedness and response-related activities due to its flexibility and easy-to-customize nature, features which also facilitate its use. IT solutions to facilitate and strengthen disease prevention and control are needed and will be developed in the coming years, and PS, as well as other tools developed under the PANDEM-2 project, can be useful prototypes to be further developed according to future needs and threats.

## Data availability statement

The original contributions presented in the study are publicly available. This data can be found here: https://github.com/pandem2/pandem-source.

## Author contributions

FO: Conceptualization, Methodology, Software, Supervision, Writing—original draft. CC: Software, Writing—review & editing. WM: Software, Writing—review & editing. JH: Supervision, Writing—review & editing. MC: Funding acquisition, Supervision, Writing—review & editing. ES: Software, Supervision, Writing—review & editing. AS: Methodology, Writing—review & editing.

## Footnotes

^1^ https://github.com/pandem2/pandem-source/blob/main/pandemsource/data/list-of-variables.csv

^2^ https://pypi.org/project/pandem-source/

^3^ https://ec.europa.eu/info/european-union-public-licence_en

^4^ https://github.com/pandem2/pandem-source

^5^ https://pykka.org/en/latest/

^6^ https://shiny.rstudio.com/
